# Mismatches between Morphology and DNA in Italian Partridges May Not Be Explained Only by Recent Artificial Release of Farm-Reared Birds

**DOI:** 10.3390/ani12050541

**Published:** 2022-02-22

**Authors:** Diego Fontaneto, Paolo Viola, Claudia Pizzirani, Stefania Chiesa, Alessandro Rossetti, Andrea Amici, Livia Lucentini

**Affiliations:** 1 Molecular Ecology Group (MEG), Water Research Institute (IRSA), National Research Council of Italy (CNR), 28922 Verbania Pallanza, Italy; diego.fontaneto@cnr.it; 2 Department of Agriculture and Forest Science, University of Tuscia, 01100 Viterbo, Italy; p.viola82@unitus.it; 3 Department of Chemistry, Biology and Biotechnologies, University of Perugia, 06123 Perugia, Italy; claudiapizzirani93@gmail.com (C.P.); livia.lucentini@unipg.it (L.L.); 4 Department of Molecular Sciences and Nanosystems, Ca’ Foscari University of Venice, 30172 Venice, Italy; stefania.chiesa@unive.it; 5 ISPRA—The Italian Institute for Environmental Protection and Research, 00144 Rome, Italy; 6 Sibillini Mountains National Park, 62039 Visso, Italy; rossetti@sibillini.net

**Keywords:** *Alectoris graeca*, artificial translocations, gamebirds, *Perdix perdix*, hybridization, introgression, mitonuclear discordance, museum, restocking, reintroduction

## Abstract

**Simple Summary:**

One of the major drivers of genetic pollution is artificial translocation, which causes hybridization and introgression. We analyzed genetic markers of Grey and Rock Partridges from collections, wild populations and farms, mostly in Italy. We documented a mismatch between morphology and DNA in the identification of some individuals, as well as hybridization between the two genera of the Grey and Rock Partridges: *Perdix* and *Alectoris*. Our results suggest that species of the two genera can hybridize in nature and that artificial translocations and releases of farm reared birds for restocking or reintroduction purposes may be only partially responsible for the DNA-morphology mismatches of Italian partridges.

**Abstract:**

Translocations and releases of farm-reared birds are considered among the major drivers of genetic pollution with consequent loss of genetic diversity in wild populations. In this study, we aimed to assess the extent of hybridization and introgression in the Italian partridges as a consequence of translocation. We surveyed two mitochondrial markers and one nuclear marker of *Alectoris* and *Perdix* from collections (museums and private collections), extant wild populations and farms. Consistent with previous studies, we found haplotypes of allochthonous species within the same genus, likely due to introductions for hunting activities. In addition, we found hybrids between *Perdix* and *Alectoris* species with genetic markers from both genera in single individuals. Such introgression was bidirectional and in both mitochondrial and nuclear markers. Counterintuitively, most of the hybrid samples came from collections before the 1950s, when large-scale translocations started, from wild populations where Grey Partridge (*Perdix* *perdix*) and Rock Partridge (*Alectoris* *graeca*) overlap in their distribution, whereas only one hybrid occurred among the farmed birds. Our results suggest that *Perdix* and *Alectoris* species can hybridize in nature and that artificial translocations and releases of farm-reared birds for restocking or reintroduction purposes may be only partially responsible for the genomic mismatches of Italian partridges.

## 1. Introduction

The Phasianidae Horsfield 1821 family includes several small to large ground-living game birds and is the richest of the seven traditional families of the Galliformes Temminck 1820 order [1,2]. Galliformes are characterized by successive events of rapid but relatively ancient radiations [3,4], creating discordance between traditional groups based on morphological assessments and the resolution of monophyletic groups by molecular phylogenies [5,6,7]. Even within Phasianidae, the two traditional subfamilies of Phasianidae Horsfield 1821 (pheasants, tragopans, junglefowl, peafowl) and Perdicinae Horsfield 1821 (partridges, Old World quails and francolins) [8] are not considered monophyletic [7,9]. 

In Europe, there are several autochthonous, native species belonging to Phasianidae, many of which have a particular economic importance as game birds for hunting activities, which frequently cause direct or indirect conservation problems for the species [10,11]. Two of such genera are *Alectoris* Kaup 1829 and *Perdix* Brisson 1760, both characterized by historical hunting exploitation [12,13,14,15,16,17] and frequent human-mediated hybridization among different species of the same genus [15,16,18,19,20,21]. The occurrence of hybridization among different species of *Alectoris* has been also described as a natural phenomenon in the Alps [22]. Indeed, these authors documented natural hybridization zones where two species get in touch spontaneously along the boundaries of the respective distribution range [22]. 

The genus *Alectoris* occurs in the southern part of the Palaearctic region with seven species. The Rock Partridge, *Alectoris graeca* (Meisner 1804), is the most widely distributed species of the genus in Italy. It is currently recognized as a polytypic species with four subspecies based on of morphological criteria [1,23]. Subsequent genetic analyses did not confirm the existence of the subspecies, underlining high genetic affinity between Italian and Balkan Rock Partridges [24]. This species, previously declared vulnerable in Europe by Bernard-Laurent and Boev [25] as well as in Italy by Peronace et al. [26] and Rondinini et al. [27], is classified as Near Threatened [28,29] and included in Annex I of the EU Birds Directive. Furthermore, it was recently classified as a Species of European Conservation Concern (SPEC) category 1, because of the continuing decline in the area of occupancy [30]. 

As frequently reported, the trend of European bird populations’ decline is related to direct and indirect anthropogenic causes, mostly due to the loss of suitable habitats [12,31,32,33,34] fragmentation and isolation of small populations [35]. Such impacts may drive species towards the extinction vortex, causing a progressive worsening of their conservation status. For these reasons, restocking and reintroduction programs were commonly considered appropriate for conservation purposes. In the past, these interventions aimed to reinforce wild relict populations but were performed without any molecular validation of species attribution of the used individuals and with no precaution against the introduction of allochthonous and potentially interfertile taxa. For instance, the diffuse release of chukar partridge, *Alectoris chukar* (J. E. Gray 1830), native to Asia, resulted in the formation of hybrids with the native Rock Partridge [15,16,21,36]. Hybridization and genetic pollution interacted with local (legal or illegal) over-hunting and environmental degradation, seriously hindering the survival of autochthonous Rock Partridge populations [32,35,37]. 

The genus *Perdix* is characterized by similar socio-economic and taxonomic issues, in part related to the intense hunting exploitation and the massive release of farm-reared birds [18]. Three species are currently attributed to this genus [38] and cover a wide area in Asia and Europe, mostly in grasslands: the Grey Partridge, *Perdix perdix* (Linnaeus 1758), is distributed in most of Europe and the western Palearctic as far as southwestern Siberia; the Daurian partridge, *Perdix dauurica* (Pallas 1811), is distributed in Russia and Mongolia; the Tibetan partridge, *Perdix hodgsoniae* (Hodgson 1857), is present in Nepal, Tibet and China. Bao et al. [38] resolved the phylogenetic relationships within the monophyletic genus *Perdix*, with *P. hodgsoniae* being the sister to the other two species. Diversification in this genus is considered related to the Tibetan Plateau uplift combined with Pleistocene glaciations [38,39]. Among these species, only the Grey Partridge is naturally present in Italy and hybridization problems were previously investigated principally between conspecifics farm-reared and wild individuals [18,40]. 

For the two Italian partridges and for other species, artificial translocations and massive releases of genetically uncontrolled farm-reared individuals into wild extant populations are known to create a series of adverse genetic effects, such as introgressive hybridization with loss of genetic diversity [18] and disruption of wild local adaptations [15,41]. Competition between wild and introduced individuals is also common, as well as the introduction of new pathogens [18]. All these factors, in addition to habitat degradation, determined an overall marked demographic decline even if the Grey Partridge is still included in the Least Concern (LC) category of the IUCN Red List of Threatened Species at both global and European scales [42,43].

The geographical distributions of several species, including those of *Perdix* and *Alectoris* genera, were deeply influenced by the cold-dry glacial periods in which the arctic Tundra covered great parts of northern and central Europe and several species moved towards the southern European areas [24,41,44]. Two glacial refugia were previously described for phasianids, located respectively in southwestern and southeastern Europe [45,46]. Glaciations, forcing distribution of animals within these refugia and limiting dispersal and gene flow between populations, shaped differentiation of genotypes and ecotypes [41]. 

However, since the second half of the 20th century, artificial translocations and massive releases of farm reared partridges were widely performed across Europe to face the rapid decline of both Rock and Grey Partridges and sustain their hunting exploitation [12,15,16,17,37,39]. These interventions, commonly performed with genetically uncontrolled individuals frequently belonging to allochthonous species or allochthonous populations of the native species, acted as an evolutionary force against the historical effect of glaciations, putting in contact taxa that have been isolated for several millennia with potentially relevant negative effects on the genetic variability of wild extant populations [15,16,37,39]. 

Morphological distinction between congeneric species is almost entirely based on characters that are non-discrete and qualitative [8,21,37,39,47], which could be often misleading especially beyond the earliest backcross generations [36]. Furthermore, Randi et al. [24] reported that plumage colour and time of feathers evolved rapidly, shaped by natural selection and/or non-genetic ecological factors. Therefore, morphology should be integrated with molecular markers to identify evolutionary independent units, in conservation biology [48]. Such DNA-based tools to identify units of conservation have already been used to allow sustainable management of *Alectoris* and *Perdix* populations [15,16,18]. 

The aim of the present study was to assess the extent of hybridization and introgression in the Italian partridges. We performed an extensive survey of the diversity of three genetic markers of *Alectoris* and *Perdix* specimens from museums, private collections, extant wild populations and farms. Indeed, assuming that native genetic diversity should be preserved in museum samples dating before the beginning of the known massive release of genetically uncontrolled birds, we aimed at assessing the magnitude of the effects and the starting time of the human-mediated hybridization in the two Italian partridge species.

## 2. Materials and Methods

### 2.1. Samples, Genetic Marker and Procedures

In most conservation-oriented genetic studies, both mitochondrial DNA (mtDNA) and nuclear DNA (nDNA) are used [15,16,20,36], offering different and complementary information on population history. Accordingly, our analyses targeted three relatively fast-evolving markers that could provide information on species identification and support evidence of hybridization and introgression. Two of the three markers were from the mitochondrial genome, namely a fragment of the protein-coding cytochrome c oxidase subunit I, *COI*, and a fragment of the non-protein-coding displacement loop, *DLOOP*; the third marker was from the nuclear genome, representing a fragment of the oocyte maturation factor mos, *CMOS*. 

Two hundred and forty-four sequences of the three selected markers of *Alectoris* and *Perdix* available in GenBank from Albania, Armenia, Canada, China, Cyprus, Denmark, Finland, France, Greece, Israel, Italy, Kashmir, Kazakhstan, Mongolia, Russia, Saudi Arabia, Scandinavia, Spain and USA were downloaded and added to the dataset composed of the new sequences obtained during this study. Newly generated sequences were obtained mainly from Italy (*n* = 959), from different individuals (i) collected from wild extant populations (*n* = 103), (ii) preserved in museums and private collections (*n* = 105) and (iii) from rearing farms (*n* = 751). Few new sequences (*n* = 16) were obtained from bird samples of unknown origins.

The overall dataset included DNA sequences from 1219 individuals for six of the seven species of the genus *Alectoris* and for all three species of the genus *Perdix* (Table S1). The species with the highest number of sequenced individuals were *P. perdix* (*n* = 857), *A. graeca* (*n* = 237), *A. chukar* (*n* = 60) and *A. rufa* (*n* = 50), whereas the other species had only from one to six sequences each. 

The overall Italian sample included 989 sequences, both as new sequences and from GenBank. On the base of our morphological assignment or species identification from GenBank sequences, 73.5% of the Italian sequences are of *P. perdix*, 22.3% of *A. graeca*, 3.4% of *A. chukar* and 0.7% of *A. rufa*.

For most birds, especially for those from collections (museums and private collections), we searched for the year when the bird was collected using labels or archives. 

Overall, 31 samples, of which 22 assigned to *P. perdix* on a morphological base, date earlier than the middle of the 20th century, before artificial translocations and massive release of genetically uncontrolled farm reared birds took place. Of the remaining collection samples, 30 are recent, dating later than the mid-20th century, whereas the other 44 samples could not be dated reliably. All the other 884 Italian samples are recent to current.

Taxidermized samples were collected under the supervision of museum staff. Both live and museum specimens were sampled with a non-invasive method, collecting just a few feathers (from three to five), avoiding any damage to the sample. DNA was extracted from a single feather or from faeces (<10 mg) modifying previous published protocol (Lucentini et al., 2010) as a new adaptation of the Wizard Genomic DNA Purification Kit (WGDPK, Promega). Faeces were included in the dataset only if the bird was seen to take flight and the samples were collected immediately. From two to four mm of feather quills or 5–10 mg of faeces were placed in a 1.5 Eppendorf tube. A solution of 500 µL of nuclei lysis solution and 120 µL of 0.5 m EDTA for each sample was prepared and placed at −20 °C until latescence (10–15 min circa depending on total volume). At this point, 17.5 µL of proteinase K (10 µg/µL) for each sample was added, firmly mixed, and 637.5 µL of the solution was added to each sample. After overnight incubation, samples were homogenized using sterile potters and incubated (65 °C, 30′). The procedure then follows the manufacturer’s instruction with the only final modification: samples showing visible pellet were re-suspended through 80 µL of DNA Rehydration Solution (Promega). Samples not showing visible pellet were re-suspended in just 50 µL of the same solution to avoid excessive DNA dilution. Tubes were incubated 10′ at 37 °C and overnight at 4 °C, then stored at −20 °C or immediately used for subsequent PCR amplifications.

A problem that we took into consideration is the cross-contamination between contemporary and museum samples and between faeces and samples having more and fresher DNA. To avoid these problems, DNA extraction was separated day by day on the basis of sample typology, avoiding processing actual and historical samples together. Furthermore, separate laboratory rooms and dedicated laminar flow hoods were used. 

PCR amplifications were performed using both already published primers or primers specifically designed for this research with published or modified protocols, as detailed below.

#### 2.1.1. Displacement Loop (DLOOP)

For the *Perdix DLOOP*, an amplification with LPPGLU (F5′-3′CACTGTTGTTCTCAACTACAGG) and H414 (R5′-3′ GGTGTAGGGGGAAAGAATGGG) primers [44] was carried out following the authors’ protocol and the mixture reported above. For some museum and faeces specimens for which the protocol did not work, a nested-PCR approach was used based on a first amplification with primers PHDL (F5′-3′AGGACTACGGCTTGAAAAGC) and SEMD621 (R5′-3′AACCTGTGAAGAAGCCCCAGA) [20] followed by a semi-nested amplification with primers PHDL and H414. The condition of these amplifications were 4′ at 94 °C followed by thirty cycles of 60″ at 94 °C, 90″ at 55 °C, and 60″ at 72 °C, followed by a final extension of 10′ at 72 °C for PHDL-SEMD621 and 4′ at 94 °C followed by 30 cycles of 45″ at 94 °C, 45″ at 55 °C, and 60″ at 72 °C, followed by a final extension of 10′ at 72 °C for PHDL-H414. For specimens belonging to *Alectoris* genus, the first amplification of *DLOOP* was performed using PHDL and H1321 (F5′-3′TAGYAAGGTTAGGACTRAGTCTT) primers [20], following the authors’ amplification conditions. Nested PCRs were then performed as reported by Fumihito et al. [49] and Barbanera et al. [20], subdividing sequences in “long fragment” (1155 bp), nested A (621 bp), and nested B (689 bp).

#### 2.1.2. Cytochrome Oxidase Subunit I (COI)

For both genera, for *COI* a first amplification was performed with Bird-F1 (F5′-3′TTCTCCAACCACAAAGACATTGGCAC) and Bird-R1 (R5′-3′ ACGTGGGAGATAATTCCAAATCCTG)) primers [50], amplifying a 678 bp fragment. We used the thermal cycle program: 5′ at 94 °C followed by 26 cycles of 30″ at 94 °C, 45″ at 52 °C, and 45″ at 72 °C, final extension of 1′ at 72 °C. Reactions were performed in a total volume of 25 µL with 50 ng of total DNA, 12.5 µL of 2x PCR Master Mix (Promega), 1 µL each of 10 µM primer and nfH_2_O to volume (25 µL). Subsequently, for museum and faeces specimens, showing difficulties in amplifying the long fragment, this amplicon was re-amplified through a nested-PCR amplification with internal primers (Bird-COI-Nes-F F5′-3′ CATAAGCTTCTGACTCCTTCCA; Bird-COI-Nes-R R5′-3′ GGGGTTTTATGTTGATGATGG). Nested-PCR cycle program was: 3′ at 94 °C followed by 29 cycles of 30″ at 94 °C, 45″ at 52 °C, and 45″ at 72 °C, followed by a final extension of 10′ at 72 °C. Nested and semi-nested protocol were based on a mixture in a total volume of 25 µL with 1 µL of first amplicon, 12.5 µL of 2× PCR Master Mix (Promega), 1 µL each of 10 µM primer and nfH_2_O to volume (25 µL).

#### 2.1.3. Oocyte Maturation Factor Mos (CMOS)

*CMOS* was obtained by a nested-PCR approach based on a first amplification with CMOS2 primers (CMOS2—F F5′-3′GCTGTGAAGCAAGTGAAGAA; CMOS2—R R5′-3′AGCCGAAGTCTCCAATCTT), specifically designed for this study, with a mixture in a total volume of 25 µL with 1 µL of first amplicon, 12.5 µL of 2× PCR Master Mix (Promega), 1 µL each of 10µM primer and nfH_2_O to volume (25 µL) amplified through the program: 2′ at 95 °C followed by 32 cycles of 30″ 94 °C, 30″ 49 °C and 2′ 72 °C, followed by a final extension of 10′ at 72 °C. If this amplification did not work, as for museum and faeces specimens, this first amplification was followed by a nested amplification with CMOS primers [4] (CMOS—F F5′-3′GCCTGGTGCTCCATCGACTGG; CMOS—R R5′-3′GCAAATGAGTAGATGTCTGCT) performed with the same program used for the first amplification, reducing cycles from 32 to 29.

#### 2.1.4. Additional Procedures

When nested amplifications were needed, amplicons were purified with EXOSAP-IT Express (Thermo Fisher Scientific) following supplier protocol, and then 1 µL of each amplicon was used in subsequent amplification. Finally, PCR products were purified using ExoSAP-IT^®^ for PCR Product Clean-Up (usb) following the manufacturer’s instructions and sequenced in forward and reverse directions by Eurofins Genomic sequencing service (http://www.eurofinsgenomics.eu, accessed on 11 November 2021).

When working with mtDNA, the risk as argued by several authors, of amplification of nuclear insertions of mtDNAs (nuclear mitochondrial DNA, NUMTs) should be considered. Following the literature, this risk was reduced by using feathers as DNA source [21,51] and carefully evaluated primers [21,52,53]. Furthermore, the presence of NUMTs was actively searched in the obtained chromatograms, looking for their signature in the form of stop codons, indels, etc. [21,51,54]. In addition, PCR products were run on 2.5% agarose gel searching for the presence of additional bands over the principal band consistent with each amplicon’s expected size and no evidence of smaller, adjunctive amplicons emerged. 

For a selection of samples that revealed potential mismatches between morphological and DNA taxonomical identifications, the different steps were repeated extracting a different feather from the same bird to confirm the reliability of the sequences and remove the possibility of contamination or mislabelling as an explanation of the apparent mismatch.

The quality filtering steps for retaining sequences for the analyses, both for the ones from GenBank and the newly generated ones, were correct translation to amino acids with no stop codons for coding markers, absence of indels for coding markers and sequence length not less than a threshold of 60% of the total length of the fragment. Only sequences that passed such quality control were retained for the analyses.

### 2.2. Phylogenetic Analyses

Sequences were aligned with MAFFT [55] with default settings. Phylogenetic reconstructions were obtained to visualize the evolutionary relationships of the sequenced DNA fragments through maximum likelihood using PhyML [56] implementing the GTR+I+G evolutionary model with four gamma categories, starting from alignments of haplotypes for each marker to remove the redundant information of identical sequences.

The approach in DNA taxonomy used to identify independently evolving units from single-locus datasets was the Automatic Barcode Gap Discovery, ABGD [57], with the alignments of all sequences for each marker as input.

The phylogenetic reconstructions and the results from DNA taxonomy were considered in comparison to the morphological identification of each individual and its origin (e.g., museum, wild, farm) in order to support qualitative inference on the potential effects of the uncontrolled release of farm-reared birds.

All the procedures were performed in compliance to European rules (Directive 2010/63/UE). The project was approved by the ethical committee of Perugia University (prot 7/2022). In particular, the approval by the ethics committee was not necessary because of the nature of some of the samples (museum individuals) and because of the non-invasive in vivo sampling method. Birds were immediately released at the same sampling site after sampling. Two feathers were collected from live animals, excluding those having a functional role, when handlingthe animals for sexing. Museum and in vivo sampling were authorized by local authorities. 

## 3. Results

### 3.1. The Dataset

The marker with the highest number of sequenced individuals was *DLOOP* (*n* = 982: 185 from GenBank and 797 new ones), followed by *COI* (*n* = 383: 55 from GenBank and 328 new ones) and *CMOS* (*n* = 131: 14 from GenBank and 117 new ones). A subset of 189 individuals had sequences from for both mitochondrial markers, 87 individuals had at least one nuclear and one mitochondrial marker, and 52 individuals had all three markers sequenced.

### 3.2. Phylogenetic Reconstructions

The phylogenetic reconstructions clearly separated two genera and all the species included in the analyses, even if for some individuals there was a mismatch between their morphological identification and one or more of the DNA sequences obtained from them. 

**Figure 1:**
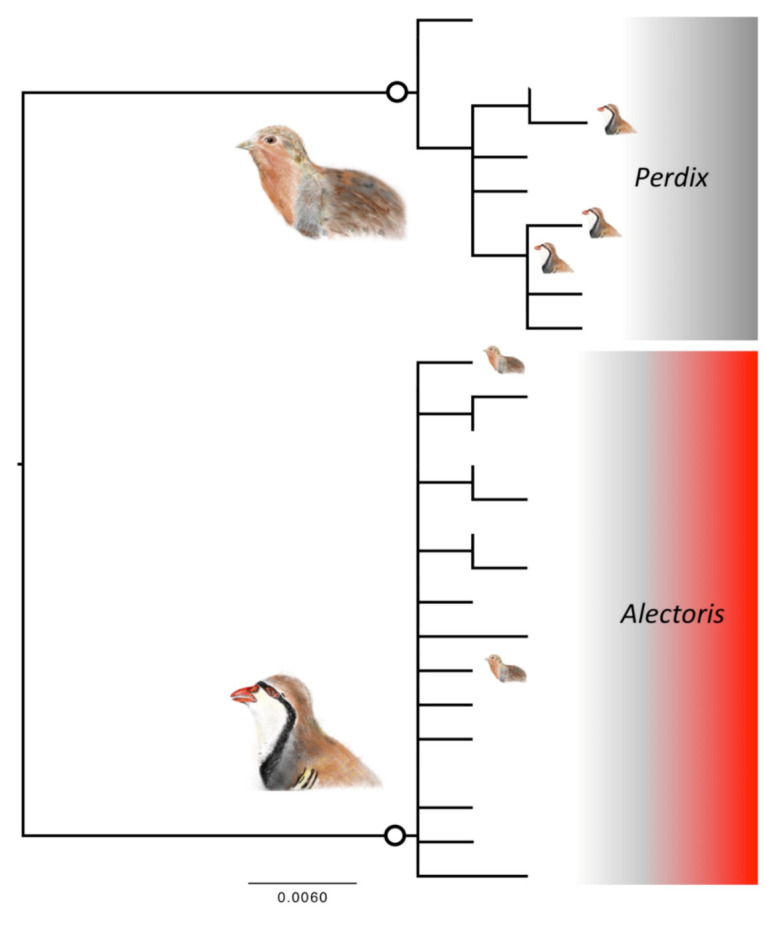
Phylogenetic relationships between haplotypes of the nuclear CMOS marker. Scale bars represent substitution rates proportional to the selected evolutionary models. The large bird icons and the colours identify the two genera, *Alectoris* (in red) and *Perdix* (in grey). The small bird icons on the tips represent cases of mismatches, with individuals of one genus falling within the species of the other genus (note that there are fewer instances than those reported in the text because the tree is at the haplotype and not at the individual level). The bordered white circled on branches identify the clades representing unique taxonomic entities according to the ABGD test on DNA taxonomy.

The 131 sequences of *CMOS* obtained for six species represented 26 sequence types, with no evidence of heterozygosity (Table S1). The sequences were not variable enough between species to allow ABGD to discriminate species within each genus, and the most likely solution of two units corresponded to the two clades representing the two genera (Figure 1).

However, the match was not perfect since eight individuals of the genus *Alectoris* fell nested within the clade of CMOS sequences of the genus *Perdix* and three individuals of Perdix fell within the clade of CMOS sequences of the genus *Alectoris* (Table 1). 

One of the 26 sequence types identified for *CMOS* (Table S1: CMOS16) was present in two *Alectoris* species and three sequence types (CMOS20, 22, 23) were present in both genera.

**Figure 2:**
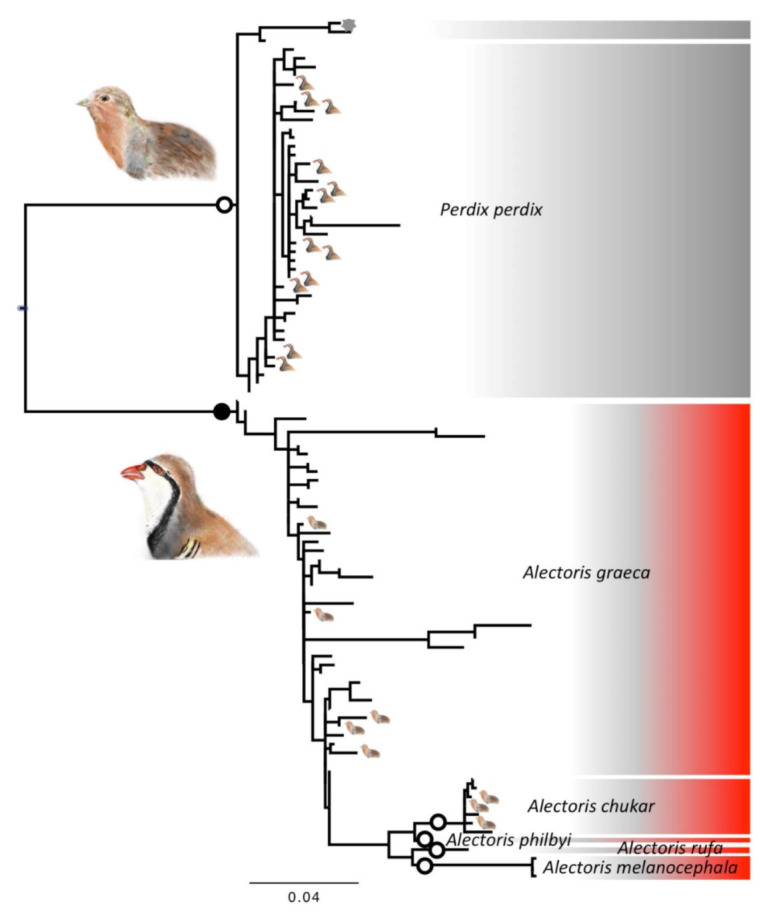
*Phylogenetic* relationships between haplotypes of COI mitochondrial marker. Scale bars represent substitution rates proportional to the selected evolutionary models. The large bird icons and the colours identify the two genera, *Alectoris* (in red) and *Perdix* (in grey). The small bird icons on the tips represent cases of mismatches, with individuals of one genus falling within the species of the other genus (note that there are fewer instances than those reported in the text because the tree is at the haplotype and not at the individual level). The grey asterisks on the tips identify cases of mismatch between species within the same genus. The separate shaded areas within each colour identify the clades belonging to morphological species, whereas the bordered white circled on branches identify the clades representing unique taxonomic entities according to the ABGD test on DNA taxonomy (in black when not monophyletic).

For *COI*, the 383 retained sequences from seven species represented 98 haplotypes. The most likely solution of ABGD revealed seven taxonomic units, broadly corresponding to the seven morphological species (Figure 2). In the case of *COI* also, there were instances of mismatch between morphological and DNA taxonomy: 20 individuals of the total 150 classified as Rock Partridges (*A. graeca*) on the base of diagnostic morphological traits fell within the *P. perdix* DNA taxonomic clade. Out of the 207 sequenced individuals with *P. perdix* morphology, one genetically fell in the *P. dauurica* clade, six in the *A. chukar* clade and six in the *A. graeca* clade (Table 1). Ten of the 98 *COI* haplotypes were present in the two species *A. graeca* and *P. perdix* (Table S1: COI17, 25, 27, 28, 33, 35, 52, 56, 60, 76).

**Figure 3:**
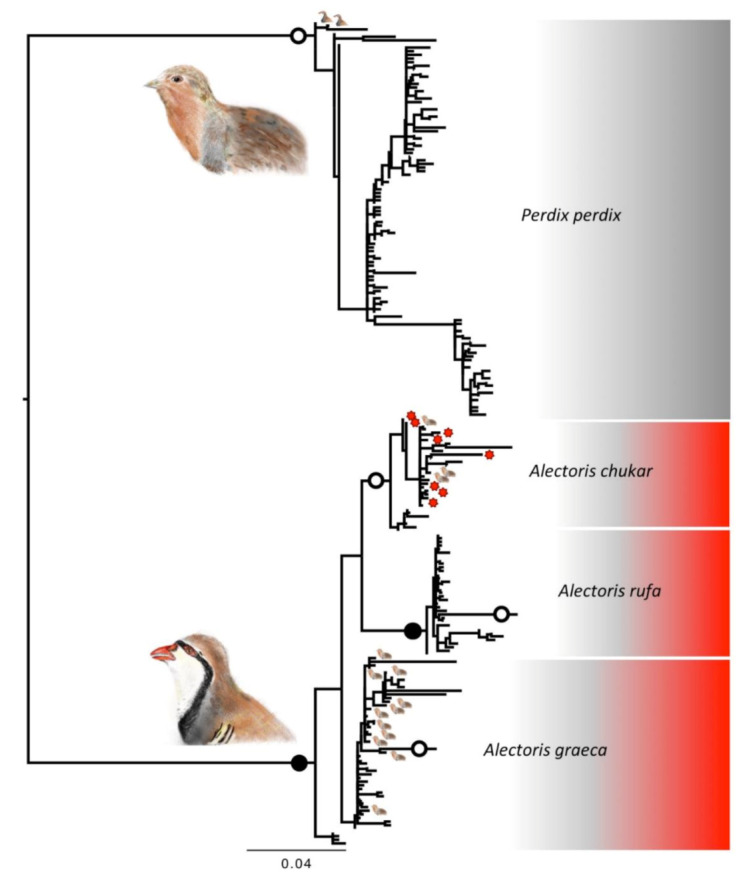
*Phylogenetic* relationships between haplotypes of DLOOP mitochondrial marker. Scale bars represent substitution rates proportional to the selected evolutionary models. The large bird icons and the colours identify the two genera, *Alectoris* (in red) and *Perdix* (in grey). The small bird icons on the tips represent cases of mismatches, with individuals of one genus falling within the species of the other genus (note that there are fewer instances than those reported in the text because the tree is at the haplotype and not at the individual level). The red asterisks on the tips identify cases of mismatch between species within the same genus. The separate shaded areas within each colour identify the clades belonging to morphological species, whereas the bordered white circled on branches identify the clades representing unique taxonomic entities according to the ABGD test on DNA taxonomy (in black when not monophyletic).

For *DLOOP*, the 982 retained sequences from four species represented 227 haplotypes. The most likely solution of ABGD revealed six taxonomic units, due to the presence of distantly related sequences within two species (Figure 3). All individuals morphologically identified as *A. chukar* clustered in one DNA taxonomic unit. For *A. graeca*, 147 individuals out of 152 clustered in one DNA taxonomic unit and another one represented a unique sequence, still phylogenetically nested within the previous unit, whereas two sequences fell within the clade of *A. chukar* and two within the clade of *P. perdix*. For *A. rufa*, 40 individuals out of 47 clustered in one DNA taxonomic unit, one individual represented a unique sequence, still phylogenetically nested within the previous unit, whereas six sequences fell within the clade of *A. chukar*. For *P. perdix*, 720 individuals out of 742 clustered in one DNA taxonomic unit, whereas 19 fell within *A. graeca* and three within *A. chukar* (Table 1). Seven of the 227 haplotypes were present in more than one species: one (Table S1: DLOOP166) was shared between *A. chukar* and *A. graeca*; four between *A. graeca* and *P. perdix* (DLOOP075, 183, 186 and 195), one (DLOOP172) between *A. chukar* and *P. perdix*, one (DLOOP169) between three species, namely *A. chukar*, *A. rufa* and *P. perdix*.

### 3.3. Spread of Mismatch between Morphological Classification and DNA Taxonomy

Overall, out of 1219 sequenced individuals, 68 (5.6%) revealed instances of mismatch between morphological classification and DNA taxonomy. For 41 of the 68 mismatches, only one sequence was available, whereas for 27 individuals two or more markers were available. 

**Figure 4:**
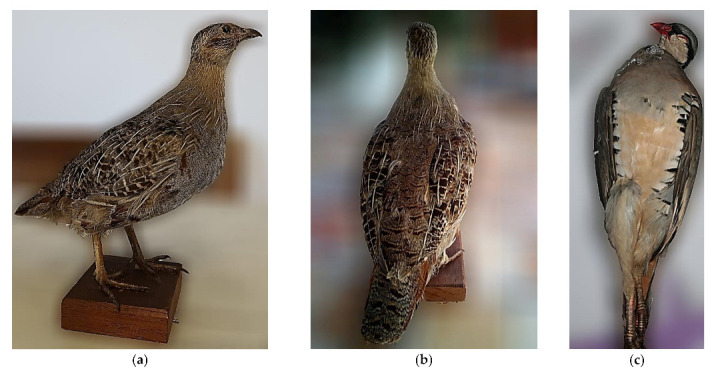
Photos of the right side (**a**) and of the back (**b**) of individual PP19_3 from Italy with unambiguous morphological features of *P. perdix* and all three genetic markers (CMOS, COI and DLOOP) of *A. chukar*. In panel (**c**) individual CO15_32 is reported, morphologically identified as *A. graeca* and DLOOP matching the morphological identification but with CMOS and COI of *P. perdix*.

Nine mismatched individuals had all three markers available; for these, only in one case the mismatch was confirmed by all three markers: individual PP19_3 from Italy (Figure 4) had unambiguous morphological features of *P. perdix*, but the three markers were unambiguous from the genus *Alectoris* and from the species *A. chukar*. Two other individuals, CO15_31 and CO15_32 (Figure 4) were morphologically identified as *A. graeca* but had *CMOS* and *COI* of *P. perdix* and *DLOOP* matching the morphological identification. The other six individuals had evidence of mismatch only from one of the three markers.

Out of the 189 individuals for which both mitochondrial markers, *COI* and *DLOOP*, were available, DNA taxonomy provided the same identification of morphology in 174 cases (92.1%); in four birds (2.1%) the disagreement with morphology was confirmed by both markers and for the other 11 individuals (5.8%), only one of the two mitochondrial markers matched the morphological species. 

Out of the overall 68 birds with evidence of discordance between morphology and DNA or between genetic markers, 63 were Italian. Of the Italian mismatched birds, six were from extant wild populations (out of a total of 104 wild birds: 5.8%), only one was from farmed birds (out of a total of 754 birds: 0.1%), 55 were from collections (out of 105 birds: 52.4%).

### 3.4. Temporal Progression of Mismatch between Morphological Classification and DNA Taxonomy

Out of the 55 mismatched birds from collections (from a total of 105), 38 had a collection date and 16 date back to earlier than the middle of the 20th century, with eight of these being even from the 19th century (Table S1). Out of the 31 collection samples from before the mid-20th century, the frequency of mismatches amounts to a surprising 51.6%.

Out of the 884 extant Italian samples and from collections dating later than the mid-1950s only 29 revealed evidence of mismatch, accounting to only 3.3%. 

## 4. Discussion

The spread of allochthonous genotypes from artificially translocated or farm-reared partridges into conspecifics or congeneric wild populations is a known pattern already found in several previous analyses [15,16,18,40,41,44,58,59,60]. Consistent with these results, we found haplotypes of the allochthonous *A. chukar* in Italian *A. graeca* (Table S1). The frequency of occurrence of such allochthonous haplotypes was 0.6%, lower than what was reported by Barilani et al. [16], but still confirming that humans have certainly played a role in the overall spread of introgressive hybridization with the introduction of allochthonous species. The genus *Perdix* allows for the occurrence of a haplotype of *P. dauurica* in an Italian *P. perdix*, collected in 1960, to also be considered consistent with the beginning of the widespread artificial translocations that widely affected Europe since the second half of the 20th century [12,15,16,17,25,37,39].

Contrary to the expectation of a recent spread of haplotypes from *A. chukar* into Italian *A. graeca*, we found it twice in samples from before the 1950s and in one case even in birds collected in the 19th century. While anecdotes [61] on sporadic releases and translocations of partridges already at the end of the 19th century, it could explain one of the evidence of haplotypes from *A. chukar* into Italian *A. graeca*, for the older case we cannot find a plausible explanation in human mediated introgression, unless such translocations started much earlier than currently known. 

Regardless of within-genus hybrids, the surprising evidence we found in our extensive survey was the spread of genotypes between different genera. Indeed, for the first time we found evidence of hybrid genotypes with genetic markers from both genera in single individuals. Moreover, such hybridization events were not rare in our survey. Out of 1219 birds in the overall dataset, at least 68 revealed evidences of a mismatch between morphology and DNA (5.6%) and for 27 of them for which we obtained two different markers, 24 showed also mismatches between markers. The two genera *Alectoris* and *Perdix* are considered distantly related and cluster in different clades, separated by at least several million years of independent evolution [2,6,47,62]. 

The extensive amount of hybridization and introgression left evidence in the patterns of (1) phenotype–genotype discordance: genetic markers of a genus were found in birds that were phenotypically unambiguously from another genus, and (2) mitonuclear discordance: sequences of mitochondrial and nuclear markers originally from two different genera were found within the same individual. Both discordances could be explained by hybridization or by incomplete lineage sorting. Unfortunately, with the available data it is not possible to accurately test which process could be more plausible [63], given the relatively low sample size of individuals from which we have all multiple markers. However, the mismatches between genera are highly unlikely to be explained by incomplete lineage sorting, given that *Alectoris* and *Perdix* are not closely related genera in the family [2,6,62]. In addition, sequences were highly and clearly different between the two genera, with uncorrected distances of 3.8% to 5.7% in *CMOS*, of 10.0% to 23.3% in *COI*, and of 15.9% to 30.3% in *DLOOP*. Thus, the discordances seem more likely due to recent or older hybridization and introgression. The fact that exactly the same haplotype is in some cases currently present in individuals of different genera makes the hypothesis of relatively recent hybridization more plausible, at least for those cases. The possibility for species of game birds of the order Galliformes to hybridize between distantly related species is not entirely novel, given that the Common Pheasant, *Phasianus colchicus*, is known to be involved in multiple numerous hybrid interactions with species of other genera [64].

In line with previous results, we also found discordance between mitochondrial markers of the same individuals: mitochondrial DNA heteroplasmy is a known phenomenon in both plants and animals [65], and it was also already described in both the Grey and Rock Partridges [21,40].

The possibility of contamination can be reliably excluded, given that for all the instances of discordance, additional feathers were obtained from the samples and the amplification procedures performed again to confirm the DNA sequences making it unlikely to find exactly the same contaminants. The unlikely scenario of birds from the museum being patched up or made to look prettier by using skin or feathers from a different species could be removed from consideration by the fact that repeated feathers confirmed the same DNA sequences.

In our case study, the spread of allochthonous genotypes from farm to wild birds, which was already highlighted as a potential problem for species management and conservation [18,58] does not seem to be the major driver of mismatches between morphology and DNA taxonomy in Italian partridges. Indeed, we found hybridization and introgression of genotypes between genera more frequently in samples from museums and collections (52.4%) and in wild birds (5.78%) than in farm-reared birds (0.1%). 

The higher propensity to find mismatches in samples stored in museums or private collections could be seen as a strong bias. Indeed, given that morphological distinction between congeneric species is almost entirely based on non-discrete characters [8,21,37,39,47], not easily detectable particularly in dead and taxidermized birds [21], wrong species identification of stored birds could provide a possible explanation for the higher likelihood of a morphological misidentification. However, this eventuality could provide only a partial explanation since an erroneous attribution to species belonging to different genera, as for instance, *Alectoris* species vs *Perdix* species, appears very unlikely. Furthermore, considering only the 25 of the 55 birds from collection for which we had multiple markers, 23 of them (92.0%) showed mismatches even between genetic markers, confirming their potential status as hybrids. 

Another potential bias favouring a higher occurrence of hybrids in collections could be related to the possibility that morphologically unusual individuals have a higher likelihood of being kept and preserved in museums or in private collections, exactly because they looked unusual, making them more likely to be actual hybrids or descendants of hybrids. 

More than the type of sample, it is the temporal progression of hybridization signature to suggest a marginal contribution of human mediated introgression in the overall spread of hybridization affecting Italian partridges. Indeed, we found the signature of hybridization between *P. perdix* and *A. graeca* more frequently (48.4%) in collection samples older than the 1950s, before artificial translocations and massive release of farm-reared birds took place, than in recent and current samples (2.3%). 

Since the two species are native to Italy and their populations reached the maximum expansion during the first half of the 20th century showing a wide contact zone along the extremes of altitude of their respective distribution range [12,37,39,66,67], our results strongly suggest that the two species may spontaneously hybridize in nature, as previously recorded for *A. graeca* and *A. rufa* by Randi and Bernard-Laurent [22], even if hybridization occurs between species belonging to different genera, as in our case. Indeed, from extant and recent samples, three *A. graeca* and three *P. perdix* with evidence of hybridization were from wild extant populations living in the Sibillini Mountains National Park (Apennines—Central Italy), where the two species are sympatric between 1300 and 1800 m. a.s.l. [68], consistently with the natural hybridization hypothesis. 

An unlikely alternative scenario should consider that translocations of farmed animals reared in promiscuous farms already occurred centuries ago.

Our results suggest that the driving mechanisms in inducing the bidirectional genetic exchange between *Alectoris* and *Perdix* could not be uniquely attributed to the process of farming, although in small artificial settings, the potential for interspecific and intergeneric mating is enhanced as previously reported for other species [69,70,71]. Usually, farming strategies are optimized for each species independently, keeping each species separated from the other to maximize the output of the farming activities [72]. The effect of separate farming is clearly visible in the fact that only one individual out of 755 farmed birds had a mismatch in our dataset. 

We acknowledge the potential weakness that out of 1219 animals for which we tested the phenotype–genotype potential mismatch, we had three sequenced markers for only 52. Thus, our survey of genetic diversity cannot provide inference on detailed patterns and processes. Yet, the main message that hybridization between genera exists and that it can be seen both in nuclear and mitochondrial markers from and towards both genera is a clear and unambiguous result.

## 5. Conclusions

In conclusion, the main result of our survey focused on Italian Grey and Rock Partridges is that genetic diversity of each of the two species passed into the other species. The recorded mismatches between genera could not be unambiguously attributed to the effect of farming these birds (almost all hundreds of farmed birds were not hybrids) but seem to be plausibly determined by hybridization in the wild. The effect of restocking and reintroduction programs of game birds was previously described as one of the main causes of the occurrence of hybrids; yet, plausible signs of hybridization were found more commonly in birds that were collected before the start of massive translocations of game birds. The highlighted mismatches between DNA and morphology in species belonging to different genera represents a call for a better understanding of the biology and management strategy for partridges, in order to avoid unexpected effects on the genotypes of the wild populations [73,74], and to not only consider restocking activities as the principal driver of genetic change in wild populations of gamebirds. 

Further research should (i) verify, in captivity, the absence of biological reproductive barriers between two genera by trying different sex-species combinations under controlled conditions and (ii) assess the frequency of natural hybrids by performing non-invasive intensive biological sampling in areas of proven sympatry. 

Pending this advancement of knowledge, it seems appropriate, in a precautionary way, to avoid forcing the two genera spatial overlapping, as for instance, releasing Grey Partridges in mountain areas where the conservation of Rock Partridge relict populations is declared a priority.

Furthermore, given the highlighted possible inconsistence between different markers, in selecting individuals for restocking or reintroduction interventions, it should be appropriate to test more than one marker. This should be encouraged especially in the case of Grey Partridge conservation programs for which the genetics is still based on mtDNA [18,39,40].

## Figures and Tables

**Table 1:** Number of individuals morphologically identified as belonging to one of the nine species included in the analyses (first column) and assigned to the different groups identified by the ABGD approach in DNA taxonomy for each of the three markers, CMOS, COI and DLOOP reported in the first row. The different groups from ABGD are named with capital letters from A to B, F, or G depending on the marker followed by the name’s initials of the species or genus with the highest number of sequences in the group: *A = Alectoris; P = Perdix; A.c. = Alectoris Chukar; A.g. = Alectoris graeca; A.m. = A. melanocephala; A.p. = A. philbyi; A.r. = A. rufa; P.d. = P. dauurica; P.p. = Perdix perdix.*

	CMOS		COI							DLOOP					
	Groups		Groups							Groups					
Morphological species	A *A*	B *P*	A *A.c.*	B *A.g.*	C *A.m.*	D *A.p.*	E *A.r.*	F *P.d.*	G *P.p.*	A *A.c.*	B *A.g*	C *A.g*	D *A.r.*	E *A.r.*	F *P.p.*
*Alectoris chukar*	5		16							41					
*Alectoris graeca*	57	8		130					20	2	147	1			2
*Alectoris magna*	1														
*Alectoris melanocephala*					3										
*Alectoris philbyi*						2									
*Alectoris rufa*							3			6			40	1	
*Perdix dauurica*		4						2							
*Perdix hodgsoniae*		3													
*Perdix perdix*	3	50	6	6				1	195	4	22				720

## Data Availability

Not applicable.

## Supplementary Materials

The following are available online at https://www.mdpi.com/article/10.3390/ani12050541/s1, Table S1: Original data for the 1219 samples included in the data set. N/A or unknown means not available information. Samples from E2 to E15 and from W2 to W30 were resumed by the Appendix of Liukkonen-Anttila et al. [44] reconstructing the sequences starting from MW.
